# The impact of high intensity training and sports on recipients of solid organ transplants: a narrative review

**DOI:** 10.3389/fspor.2024.1439399

**Published:** 2024-09-24

**Authors:** A. Kayeye, I. Triantafyllou, S. Mathur, T. Janaudis-Ferreira

**Affiliations:** ^1^ School of Physical and Occupational Therapy, McGill University, Montreal, QC, Canada; ^2^ School of Physiology and Pneumology, Aristotle University, Thessaloniki, Greece; ^3^ School of Rehabilitation Therapy, Queen’s University, Kingston, ON, Canada; ^4^ Canadian Donation and Transplantation Research Program, Edmonton, AB, Canada; ^5^ Respiratory Epidemiology and Clinical Research Unit, Centre for Outcomes Research and Evaluation, Research Institute of the McGill University Health Centre, Montreal, QC, Canada

**Keywords:** high intensity exercise, sports, athletic performance, swimming, cycling, running, transplantation, transplant recipients

## Abstract

**Objectives:**

High intensity exercise in individuals post solid organ transplant (SOT) remains a largely understudied phenomenon, with potential risks and benefits. Additionally, the optimal training protocols are still unclear. This narrative review aimed to explore the impact of high-intensity exercise training and strenuous sports on solid organ transplant recipients (SOTRs).

**Methods:**

We conducted a narrative review of intervention studies of any design that included high-intensity exercise training and cross-sectional studies of strenuous sports and activities. Additionally, we reviewed individual reports documenting post-SOT performance at highly competitive or physiological levels. We used MEDLINE to search for relevant articles followed by a manual search for additional articles. Data were extracted and results were summarized.

**Results:**

High-intensity and strenuous exercise appears to be safe among stable SOTRs. High-intensity protocols consistently demonstrated improvements in VO2peak and a reduction in coronary artery disease prevalence, though findings related to body composition, health-related quality of life outcomes, and cardiovascular exercise variables were inconsistent. Pre-transplant athletes showcase notable achievements and physiological adaptations post-transplantation, highlighting the capacity for athletic performance among this population. However, caution is warranted in interpreting the findings from these studies due to limitations in generalizability and other methodological limitations.

**Conclusion:**

As evidenced by current literature, high intensity exercise emerges as a promising exercise method for safely improving various physiological parameters, and reducing the prevalence of coronary heart disease in SOTRs. It can induce similar or greater effects to moderate intensity exercise, however follow-up studies indicate low retention. Further research of higher methodological rigor is warranted in this field to advance understanding, and to guide evidence-based practice.

## 1 Introduction

Solid organ transplant (SOT) allows for an enhanced quality of life for individuals with end-stage organ failure (1). However, Immunosuppressive medications used for post-transplant management have several side effects including, increased infection risk, reduced cardiopulmonary function and muscle strength, increased osteoporosis and risk fracture, and increased obesity prevalence (2, 3).

Furthermore, depression affects up to 60% of solid organ transplant recipients (SOTRs) and is associated with increased medication non-compliance and graft loss (4). These negative side effects can be counteracted with exercise as it has been shown to lower psychological stress, increase cardiovascular capacity, and increase bone mineral density in the general and osteoporotic population (5, 6). While moderate intensity exercise offers clear benefits in the factors related to common health risks. The safety and efficacy of high intensity exercise programs remains understudied and has sparked considerable debate and deliberations within the transplant community (7, 8). High-intensity training, defined as a workload of 85% or more of an individual’s VO^2^max, can yield comparable or superior results compared to moderate-intensity exercise (workload of 60%–80% of an individual's VO^2^max) within the general population (9–11). High intensity training has demonstrated enhancement of cardiovascular function, bone strength, immune function, body composition, and physical capacity in patients with coronary artery disease, heart failure, and immune system dysregulation (12). High intensity interval training (HIIT) is associated with greater enjoyability, and time efficiency compared to moderate intensity exercise. It has prompted researchers and clinicians to explore its potential application in the rehabilitation and fitness regimens of SOTRs (9, 12). However, HIIT (exercise involving short bursts of intense exercise) and strenuous exercise (activities lasting over 90 min with high cardiovascular demands) have immunosuppressive effects in healthy populations which may pose a potential risk for SOTRs (13). The combination of their immunosuppressive medication with the additional immunosuppressant effects of exhaustive exercise could heighten susceptibility to infections, presenting a significant concern (14).

Some SOTRs embrace vigorous exercise regimens, despite the challenges they may entail. Organizations such as the World Transplant Games Federation, Canadian Transplant Games and Transplant Sports, emphasize the importance of maintaining an active and healthy lifestyle post-Transplant. They promote participation in sports and activities that can be physically demanding for some SOTRs (7, 15). Evidence from surveys conducted among participants in the Canadian Transplant Games reveals a strong desire for structured training programs. A significant proportion of participants expressed interest in general conditioning programs to enhance physical preparedness (15). In response to these findings, the development of tailored pre-competition training programs emerges as a potential strategy to incentivize participation and facilitate higher training intensities.

However, these programs still need to be developed and undergo testing to determine their efficacy and safety. For example, in a self-perspective written by a kidney transplant recipient, they describe their uncertainty in which training regimens to undertake in order to begin competitively swimming (16). Establishing comprehensive and personalized training guidelines can offer valuable support to transplant recipients aiming to engage in athletic pursuits, ensuring both their safety and optimal performance.

Given the nuanced nature of SOT, which involves variables such as the specific organ transplanted, the recipient's pre-transplant exercise capacity level, the complexity of immunosuppressive drug protocols, and the presence of comorbidities, participation in high-intensity exercise protocols warrants careful scrutiny. This narrative review aims to explore the impact of high-intensity exercise protocols and strenuous sports on SOTRs, while prioritizing safety. We conducted a comprehensive review of the physiological and health related quality of life, clinical outcomes, and safety considerations associated with high intensity training interventions or strenuous sporting activities among this distinctive population.

## 2 Methods

A systematic search of MEDLINE along with a manual search from inception to January 18, 2024 was conducted. The key words and subject headings used are presented in Table 1. We included intervention studies of any design that included high-intensity exercise training in SOTRs. High-intensity exercise was defined as physiological intensities of vigorous to maximal effort (HRR >60 bpm, HRmax > 77%, VO2max > 64%, RPE > 14) (17). High intensity interval training (HIIT) was also considered and is typified by periods of short bursts of intense exercise, followed by rest periods. We also included cross-sectional and case studies of strenuous sports and activities performed by SOTRs. Strenuous sports were defined as activities lasting more than 90 min involving challenging tasks such as, steep altitude accession, prolonged physical exertion, or high cardiovascular demand. Outcomes of interest were: VO2peak, endothelial function and inflammatory markers, blood pressure, cardiopulmonary exercise variables, physical activity and sedentary time, body composition, health related quality of life, immunological response, inflammation and kidney response, and implications of strenuous altitude ascension.

**Table 1:** Medline keywords.

Database	Search terms
Medline	Organ transplant recipient*.mp or transplant recipients/or post heart transplantation.mp or after heart transplantation.mp or post liver transplantation.mp or after liver transplantation.mp or post kidney transplantation.mp or after kidney transplantation.mp or post pancreas transplantation.mp or after pancreas transplantation.mp or post lung transplantation.mp or after lung transplantation.mp and exp running and cycling.mp or swimming/or trialtoln.mp or marathon.mp and high intensity exercise*.mp or high intensity interval training.mp. or High-Intensity Interval Training/or Para-Athletes/or Athletes/or athlete*.mp. or exp Athletic Performance/or athletic performance

## 3 Findings and discussion

**Figure 1:**
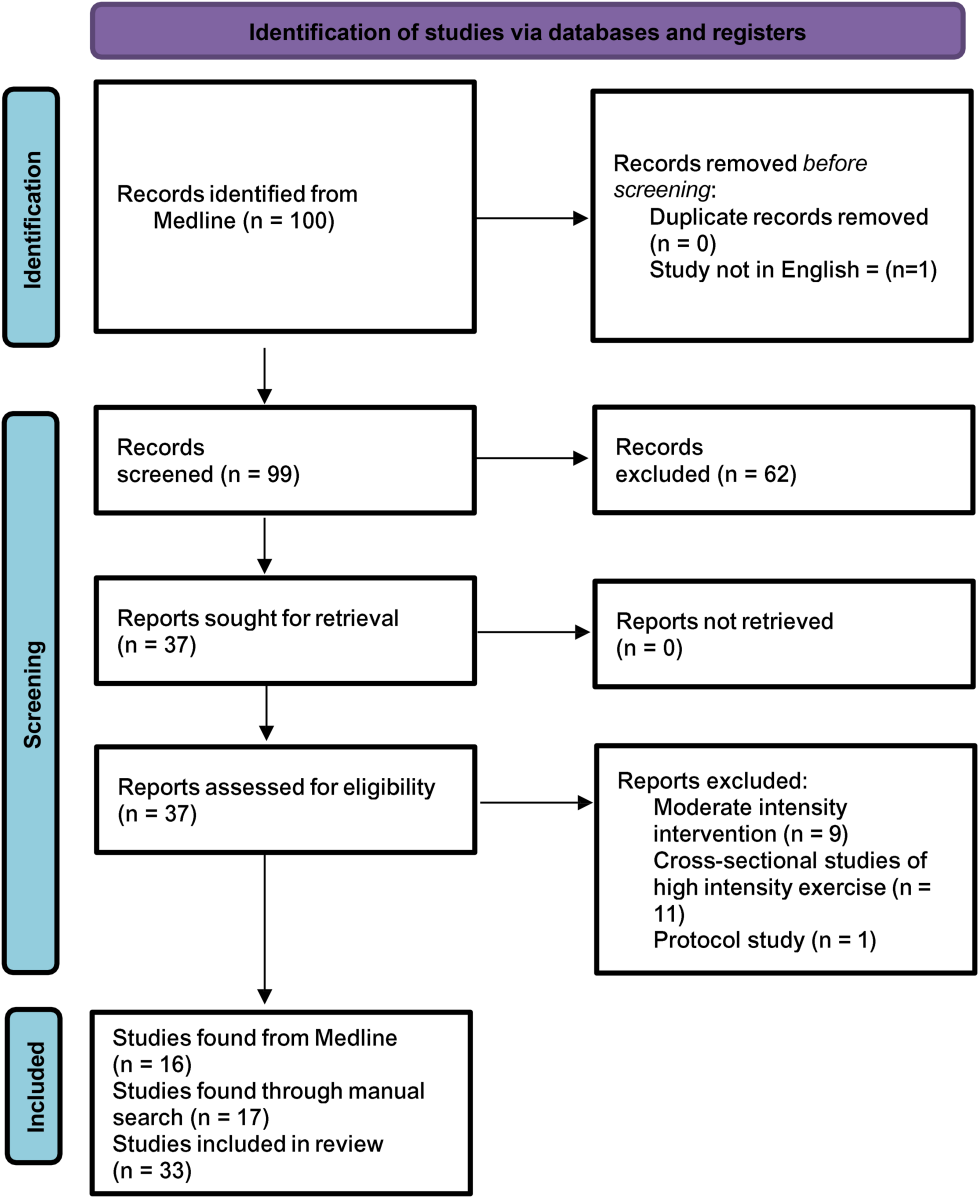
Prisma flow diagram.

The search from MEDLINE produced 100 articles (Figure 1) with no duplicates, and 37 full text articles were reviewed by 1 author (A.K) for eligibility in which 16 met the criteria. An additional 18 articles were identified through manual searches, resulting in 34 articles in total. Overall, 17 high intensity (Table 2), 6 strenuous sports and activities (Table 3) and 9 individual reports of post-SOT performance at highly competitive or physiological levels (Table 4) were retrieved.

**Table 2:** High intensity interval training.

Study/year	Country	Study objective	Organ group	Exercise Group (sample size/characteristics)	Control group (sample size/characteristics)	Time post transplant	Exercise intervention	Control arm	Outcomes studied	Main findings	Adverse effects	Study limitations	Immunosuppressive therapy
Haykowsky et al. (18)	Canada	To Examine the effects of aerobic and strength training versus no-training on VO2peak, exercise left ventricular systolic function, peripheral vascular function, lean tissue mass and maximal strength in clinically stable HeartTR	Heart	*N* = 21 Age 59 ± 11 4 Female 17 Male	*N* = 22 Age 57 ± 10 4 Female 18 Male	EG (years) s4.4 ± 3.3 CG (years) 5.4 ± 4.9	12 weeks Cycle ergometer and treadmill First 8 weeks Aerobic training (5 days/week): 60%–80% of VO2peak for 30–45 min Strength training (2 days/week): 1–2 sets of 10–15 repetitions performed 2 days/week at 50% of maximal strength. Final 4 weeks Continuous aerobic training (3 days/week): 80% of VO2peak for 45 min HIIT (2 days/week): 30 s at 90%–100% of BL peak power output followed by 60 s rest. Strength training (2 days/week): Same as above	Usual activities of daily living	Primary outcome: change in VO2peak Secondary outcome: exercise left ventricular systolic function, peripheral vascular function, lean tissue mass and maximal strength	12 weeks of SET resulted in a significant increase in VO2peak, leg and total lean tissue mass, leg-press and chest-press maximal strength without altering exercise LV systolic function or brachial artery endothelial function in clinically stable HeartTx recipients.	None Reported	Effect of 12 weeks of SET on peak LV systolic function is unknown. Did not assess resting or exercise diastolic function Lipid profiles were not measured Lack of active control group	Calcineurin inhibitor, Antiproliferative agent Corticosteroids
Hermann et al. (19)	Denmark	Examine whether high intensity aerobic exercise improves peak oxygen uptake and endothelial function in HeartTR	Heart	*n* = 13 Age 53 ± 11 1 Female 12 Male	*n* = 13 Age 47 ± 18 3 Female 10 Male	EG (years) 6.8 ± 4.0 CG (years) 7.0 ± 5.5	8 weeks Cycle ergometer and staircase running 3x/week for 42 min HIIT: 4 min: 80% VO2peak with 30 s rest 2 min: 85% VO2peak with 30 s: 90% VO2peak with 30 s rest	Assumed to be sedentary	Primary outcome: VO2peak Secondary outcome: FMD of the brachial artery, blood pressure, markers of inflammation and natriuretic peptides	High intensity aerobic exercise training in stable HeartTx recipients increased maximal oxygen uptake and improved endothelial function. Exercise was further associated with a reduction in systolic blood pressure and reduced plasma levels of proatrial natriuretic peptide as well as high sensitive C-reactive protein.	None reported	Study was not powered to detect a change in biomarker. Patients were treated with vasodilators which could interfere with the effect of exercise on FMD. Patient-initiated lifestyle interventions apart from exercise may have been initiated during the study period. Randomization to the exercise group might have resulted in behavioral changes influenced by group assignment.	Calcineurin inhibitor Antiproliferative agent Corticosteroids
Nytroen et al. (20)	Heart	To evaluate if HITT is applicable and safe in HeartTx recipients, its effect on VO2peak, and central and peripheral mechanisms behind a potential VO2peak increase.	Heart	*n* = 24 Age 48 ± 17 16 Male 8 Female	*n* = 24 Age 53 ± 14 17 male 7 Female	EG (Years) 4.3 ± 2.4 CG (Years) 3.9 ± 2.1	1 Year Treadmill 3x/week for 25 min HIIT: 4 × 4 min at 85%–95% of HRmax with 3 min active recovery periods.	Basic general care	Outcomes: VO2peak, HRQOL, Muscle strength, Muscular exercise capacity, Inflammatory myocardial markers	HIIT is applicable and safe in HeartTx recipients. HIIT significantly improved VO2peak, as compared to no changes in the control group. There were also significant improvements in muscular exercise capacity, a decrease in resting HR, an increase in HR reserve and increase in VEmax, without any changes in parameters of systolic and diastolic myocardial function or parameters of inflammation.	None reported	Inclusion criteria and type of intervention may have led to a selection bias (high baseline VO2peaks) Study population was relatively small and lacked reasons for excluding patients from the initial screened population. Over 90% of the patients were still on low-dosage steroids, and based on their negative influence on muscle function, this may have affected the results. Since the CG did not undergo another exercise it can only be stated that HIIT is an effective and safe form of exercise in this population.	Cyclosporine Tacrolimus Everolimus Mycophenolate Prednisolone
Nytrøen et al. (50)	Norway	To evaluate if high-intensity interval training (HIIT) reduces the progression of CAV among HeartTx recipients.	Heart	*n* = 20 Age 51 ± 17 7 Female 13 Male	*n* = 23 Age 53 ± 15 7 Female 16 male	EG (years) 4.1 ± 2.4 CG (years) 3.9 ± 2.1	1 Year Treadmill 3x/week for 25 min HIIT: 4 × 4 min at 85%–95% of HRmax with 3 min active recovery periods.	Basic general care	Atheroma volume,. Maximal intimal thickness, Qualitative plaque progression, Inflammatory activity	HIIT, among stable HeartTx recipients, resulted in a significantly impaired rate of quantitative CAV progression. Together with statins and immunosuppressive therapy, HIIT may be included as a treatment option in the follow-up of HeartTx recipients.	None reported	Most baseline IVUS parameters were numerically higher in the control group than in the HIIT group. May be a confounding factor affecting the results.	Cyclosporine Tacrolimus Everolimus Mycophenolate Prednisolone
Rustad et al. (21)	Norway	Investigated whether HIIT improved cardiac function and exercise capacity in stable heart transplant recipients by use of comprehensive rest- and exercise-echocardiography and cardiopulmonary exercise testing.	Heart .	*n* = 24 Age 56 (20–72) 8 Female 16 male	*n* = 24 Age 58 (19–71) 7 Female 17 male	EG (years) 5 (1–8) CG (years) 4 (1–7)	1 Year Treadmill 3x/week for 25 min HIIT: 4 × 4 min at 85%–95% of HRmax with 3 min active recovery periods.	Basic general care	Primary outcome: VO2peak Secondary outcomes: left and right ventricular systolic and diastolic function	HIIT is applicable and safe in HeartTx recipients despite denervation and improves exercise capacity without altering cardiac function significantly.	None reported	The results are most likely representative of stable HeartTx recipients in general. LV systolic and diastolic function were not assessed during maximal bicycle exercise	Cyclosporine Tacrolimus Everolimus Mycophenolate Prednisolone
Yardley et al. (22)	Norway	The main aims of this study were to present the results from a 5 year follow-up evaluating whether the HITT group was motivated to continue with HIT after the intervention period ended and whether they retained their superior physical capacity in the long-term.	Heart	*n* = 21 Age 47 ± 18 14 Male 7 Female	*n* = 20 Age 52 ± 15 14 Male 6 Female	EG (years) 4 ± 2 CG (years) 4 ± 2	1 Year Treadmill 3x/week for 25 min HIIT: 4 × 4 min at 85%–95% of HRmax with 3 min active recovery periods.	Basic general care	Outcomes: Physical activity, Exercise variables, Muscular exercise capacity, Physical capacity, Body composition, Metabolic profile, HRQoL, Depression and Anxiety, CAV	A moderate level of exercise and intensity is insufficient to maintain the higher VO2peak levels that were achieved after the HIIT intervention. A 5-year follow-up revealed a moderate activity pattern in both the HIIT group and the control group. Intermittent periods of HIIT are likely to be necessary to maintain the previously achieved high VO2peak levels. HIIT can reduce the development of anxiety symptoms in the long-term. Development of CAV, muscular exercise capacity and chronotropic responses, the long-term effects after HIIT were neutral between the groups.	None Reported	Small sample size Non-exercising control group	Cyclosporine Tacrolimus Everolimus, Mycophenolate/Azatioprine Prednisolone
Nytrøen et al. (23)	Norway	To determine the effects of HIIT in *de novo* heart transplant recipients (1 year follow-up)	Heart.	*n* = 37 Age 50 ± 12 Female 9 28 Male	*n* = 41 Age 48 ± 15 Female 12 29 Male	11 weeks for both groups	9 month Cycle ergometer 1–3x/week for 40 min HIIT: 2–4 min intervals at 85%–95% of peak effort 1 resistance training session per week	9 months Cycle ergometer 2–3x/week for 40 min Moderate Intensity Continuous Training: 60%–80% of peak effort) 1 resistance training session per week	Primary outcome: VO2peak Secondary outcome: tolerability,safety, adverse events, isokinetic muscular strength, body composition, HRQOL, left ventricular function, hemodynamics, endothelial function, biomarkers	HIIT was a safe, efficient method of exercise in *de novo* HTx recipients. Compared with MICT, HIIT resulted in significantly larger increases in the Vo2peak, anaerobic threshold, peak expiratory flow, and muscular exercise capacity. Only the HIIT group showed significant improvements in the resting a-v O2 diff and O2 pulse.	None Reported	Small sample size. Evaluated variables were collected at rest. Using O2 pulse as a surrogate for stroke volume. Only supervised exercise was recorded. Administering a quadriceps muscle biopsy would have provided better insights.	Cyclosporine Tacrolimus Everolimus Prednisolone Mycophenolate
Rolid et al. (24)	Norway	To investigate the effect of HIT vs. MICT on HRQoL in *de novo* recipients.	Heart	*n* = 37 Age 50 ± 12 Female 9 28 Male	*n* = 41 Age 48 ± 15 Female 12 29 Male	12 months for both groups	9 month Cycle ergometer 1–3x/week for 40 min HIIT: 2–4 min intervals at 85%–95% of peak effort 1 resistance training session per week	9 months Cycle ergometer 2–3x/week for 40 min Moderate Intensity Continuous Training: 60%–80% of peak effort) 1 resistance training session per week	Change in HRQoL	In patients who had recently undergone HeartTx, the Physical Component Scores improved significantly. There were no differences in HRQoL between patients allocated to HIIT or MICT, except on the Role Emotional subscale where the HIIT group had a significantly higher score.	None Reported	The high baseline HRQoL scores may reflect an above average healthy population and (limits generalizability). A disease-specific HRQoL questionnaire could have been more sensitive to detect differences between groups.	Cyclosporine Tacrolimus Everolimus Prednisolone Mycophenolate
Rolid et al. (25)	Norway	To determine whether the effect of early initiation of HIIT VO2peak persisted for 2 years postintervention (3 year follow-up)	Heart	*n* = 28 Age 53 ± 11 7 Female 21 Male	*n* = 34 Age 51 ± 14 8 Female 26 Male	36 months for both groups	9 month Cycle ergometer 1–3x/week for 40 min HIIT: 2–4 min intervals at 85% to 95% of peak effort 1 resistance training session per week	9 months Cycle ergometer 2–3x/week for 40 min Moderate Intensity Continuous Training: 60%–80% of peak effort) 1 resistance training session per week	Primary outcome: change in VO2peak Secondary outcomes: muscle strength, body composition, heart rate response, health-related quality of life, daily physical activity,biomarkers, and heart function	Early allocation to HIIT post-HeartTx did not result in sustained improvement at 3-year follow-up in VO2peak, compared to allocation to MICT. However, there was a significant difference between the groups in muscle endurance and AT in favor of the HIIT group. A high proportion in both groups still were performing PA for at least 30 min daily, while HRQoL scores were high and comparable to those in the age- and sex- adjusted general population. Early supervised cardiac rehabilitation may have sustainable effects on the daily PA after HeartTx. However, only a few participants continued with HIIT after the supervised intervention.	None Reported	The limited number of patients may have hindered the detection of subtle between-group differences. lack of a non exercising control group. High level of PA documented by self-report and activity monitors in both groups might be a result of social desirability Inclusion criteria may have influenced the low rates of participants with symptoms of depression and anxiety Single-center design of the study 3-year follow-up of the HITTS study limits the generalizability compared to a multicenter	Cyclosporine Tacrolimus Everolimus Prednisolone Mycophenolate
Ulvestad at el. (26)	Norway	To evaluate the efficacy of high-intensity endurance and strength training in LungTx recipients from 6 months to 5 years after transplantation	Lung	*n* = 25 age 52.3 ± 11.9 14 Female 11 Male	*n* = 29 age 51.1 ± 13.5 13 Female 16 Male	EG (months) 30.2 ± 16.6 CG (months) 26.6 ± 15.7	20 weeks Treadmill 3x/week for 1h High Intensity interval training: 4 min at 85%–95% HRM with 2 min recovery Strength training: 3 sets of 6–12 repetitions	followed hospitals recommendation-s	Primary outcome: change in VO2peak Secondary outcomes:pulm-onary function, muscular strength, HRQOL and physical function	Mixed-mode HIIT improved muscular strength but not VO2peak after LungTx. Strength training in particular appears to be beneficial for patients after LungTx. High- intensity exercise training initiated early (<2 years) after transplantation with acceptable adherence appears to have beneficial effects on VO2peak following LungTx.	None Reported	Little participant adherence to exercise protocol (46 interruptions) Small sample size	Tacrolimus Cyclosporine Mycophenolate mofetil Prednisolone
Ulvestad et al. (27)	Norway	To assess the effects of high-intensity training (HIIT) on body composition, bone health, and physical activity in lung transplant patients.	Lung	*n* = 22 age 51.6 ± 12.3 13 Female 9 Male	*n* = 29 age 51.1 ± 13.7 13 Female 16 Male	EG (months) 32.5 ± 16.6.2 CG (months) 26.7 ± 15.9	20 weeks Treadmill 3x/week for 1h High Intensity interval training: 4 min at 85%–95% HRM with 2 min recovery Strength training: 3 sets of 6–12 repetitions	followed hospitals recommendation-s	Outcomes: Lean body mass, bone mineral density of lumbar spine and trabecular bone score, physical activity and sedentary time	HIIT improved some measures of bone health and body composition. While trabecular bone score increased and subcutaneous adipose tissue decreased, there were no improvements in lean body mass, fat body mass, bone mineral density, or physical activity level.	None Reported	8 participants missed the follow-up. Parent study was powered for (VO2peak) and not for secondary outcomes meaning sample size may have been too small to detect significant differences in body composition and BMD. The HIIT group had suboptimal adherence to the training regime. Testing for several outcomes amplifies the probability of a false-positive finding.	Tacrolimus Cyclosporine
Dall et al. (28)	Denmark	To compare the effect of HIIT versus CON in stable HeartTx recipients.	Heart	Crossover study *n* = 16 age 51.9 4 Female 12 Male		(years) 6.4 years	12 weeks Cycle ergometer 3x/a week for 16 min High Intensity interval training: 4–1 min intervals at >80% of VO2peak, with 2-min active rest period 5 month washout Continued moderate exercise (CON) 45 min: 60% to 70% of VO2peak.		Primary outcome: VO2peak Secondary outcomes: blood pressure,HRpeak, HRrest, heart rate reserve, HRrecovery and workload.	Superior effect of HIIT over CON on VO2peak. Moreover, only HIIT improved HRpeak and HRreserve whereas both HIIT and CON improved HRrecovery. There was a marked loss of effect after 5 months, emphasizing the need for repeated intervention programs and seeking lifelong change in exercise habits.	None reported	Small sample size Convective and diffusive oxygen conductance and the skeletal muscle oxygen utilization were not examined Cardiac function was not measured directly in relation to the study Study was not powered to detect change in secondary findings	Cyclosporine Mycophenolate mofeti Steroids
Dallet al. (29)	Denmark	To compare the effect of HIIT vs continued moderate training (CON) on vascular function, biomarkers and health-related quality of life (HRQoL) in HTx recipients.	Heart	Crossover study *n* = 16 age 51.9 4 Female 12 Male		EG (months) 32.5 ± 16.6.2 CG (months) 26.7 ± 15.9	12 weeks Cycle ergometer 3x/a week for 16 min High Intensity interval training: 4–1 min intervals at >80% of VO2peak, with 2-min active rest period 5 month washout CON 45 min: 60% to 70% of VO2peak.		Primary outcome: VO2peak Secondary outcomes: endothelial function arterial stiffness, biomarkers, HRQoL and markers of anxiety and depression.	Both exercise programs decreased markers of anxiety and increased the physical component in HRQoL, whereas no effects were seen for endothelial function, vascular stiffness, markers of inflammation, glucose metabolism or HOMA index. The effects achieved were lost during the 5- month washout period, highlighting the need for lifelong participation in exercise programs to maintain the positive effects of HRQoL.	None reported	Small sample size Not powered to detect changes in endothelial function, HRQoL or biomarkers When compared with the entire historic population of transplant recipients in the Copenhagen program, the patients included in this study were older and a larger proportion of them were female (33% vs 24%).	Cyclosporine Mycophenolate mofeti Steroids
Cappelle et al. (30)	Belgium	To determine whether HIT in long-term stable recipients of 4 different solid organ types can safely establish an improvement in physical performance. Additionally, the potential midterm sustainability effect of HIIT in TR was investigated.	12 Heart 7 Lung 8 Liver 15 Kidney	*n* = 42 Age 41.4 ± 11.1 years; Female 11 Male 31		(years) 6.4 years	6 months Cycle ergometer 3/week for 30 min 2 min: 85%–90% (>6 METs)	.	Primary outcome: Exercise capacity Cardiovascular parameters BMI and Body weight Secondary outcome: cycle 25.7 km and reach Mont Ventoux summit	A 6-month HIT intervention in stable long-term SOTRs safely improved exercise capacity to a level needed for strenuous physical exercise. For recipients of each organ type, the training intervention produced a benefit in exercise performance and/or cardiovascular health or body composition, although the nature of the effect varied between organ types. These improvements in exercise capacity were, however, not sustained at 6 months follow-up, suggesting a need for continued exercise training. Additionally, 1 participant was able to reach the Mont Ventoux summit.	None Reported	Observational, non controlled study Small sample sizes for the organ-specific subgroups Study design and nature of the exercise intervention warrant careful comparison with the literature. Selection criterion of a long-term stable graft function, significant spontaneous recovery of physical performance and baseline predVO2max can be associated with selection bias The combined individual/group program with motivational coaching toward an ambitious goal, can be associated with participation bias Only 14 of 42 participants, exercise capacity were assessed during follow up	Azathioprine Mycophenolate mofetil Cyclosporine Tacrolimus Steroids
Rafique et al. (31)	Norway	To evaluate the short-term effects of HIIT on CAV in *de novo* HeartTx recipients as assessed by OCT	Heart	*n* = 23 Age 47 [40–56] Female 6 Male 17	*n* = 33 Age 54 [42–57] Female 6 Male 26	6–8 weeks for both groups	6 months Cycle ergometer 58 sessions each 25 min HIIT: 4 × 4-min at 85%–95% of the peak effort with 3 min of active recovery Strength training	6 months Cycle ergometer 58 sessions each 25 min Moderate Intensity Continuous Training: 60%–80% of the peak effort. Strength training	Primary outcome: change in the mean intima area Secondary outcomes: Change in the mean lumen area, changes in plaque burden, change in MIT and changes IMR.	HIIT did not significantly reduce CAV progression the first year after HeartTx. CAV develops early after HeartTx and that the mean intima area increased by 25% within the first year.	None Reported	The statistical power analysis was performed for the outcomes in the main study and not the current substudy. Small sample size A type 2 error can be suspected, particularly for the primary endpoint since the numerical change was twofold higher in the MICT group. The follow-up period was short, and a long-term follow- up may have presented different results Patient-related interventions such as differences between groups in self-initiated exercise and dietary habits may have affected the outcomes. The importance of intracoronary imaging for detection of early CAV has recently been questioned	Cyclosporine Mycophenolate
Billany et al. (32)	U.K	To assess the recruitment, retention and adherence to HIIT and MICT programs in KidneyTx recpeints	Kidney	HIIT A Age 41 ± 14 *n* = 8 5 Female 3 Male HIIT B Age 51 ± 11 *n* = 8 2 Female 6 Male	MICT Age Age 52 ± 11 *n* = 8 1 Female 7 Male	More than 12 weeks for both groups	8 weeks Cycle Ergometer 3x/week HIIT A: 30 min of 4-, 2-, and 1-min intervals; 80%–90% watts at V̇O2peak with 2 min active rest HIIT B: 30 min of 4 × 4 min intervals at 80%–90% V̇O2peak with 3 min rest and 5 min active stage	8 weeks Cycle Ergometer 3x/week for 40 min MICT: 50%–60% V̇O2peak	Primary outcome: recruitment, retention, and intervention adherence and acceptability. Secondary outcomes: cardiorespi- ratory fitness, body composition, haemodynamic parameters, physical func- tion, habitual physical activity, markers of cardiovascular risk, inflammation, and immune function	HIIT and MICT performed on a cycle ergometer could be considered safe and feasible in KidneyTx recipients Also, both HIIT and MICT may be useful prescriptions to reduce CVD burden in KidneyTx recipients.	None reported	Small sample size unable to detect statistical changes due to inadequate powering heterogeneity in participant characteristics at baseline with respect to gender, blood pressure, cardiorespiratory fitness, and transplant vintage Of the 20 participants who completed the intervention, only eight achieved the required exercise intensity in watts.	Calcineurin Inhibitors Steroids
Hutchinson et al. (33)	U.K	To explore the physiological and immunological impact of 8-weeks of HIIT and MICT in kidneyTx recipients.	Kidney	HIIT A Age 41 ± 14 *n* = 8 5 Female 3 Male HIIT B Age 51 ± 11 *n* = 8 2 Female 6 Male	MICT Age Age 52 ± 11 *n* = 8 1 Female 7 Male	More than 12 weeks for both groups	8 weeks Cycle Ergometer 3x/week HIIT A: 30 min of 4-, 2-, and 1-min intervals; 80%–90% watts at V̇O2peak with 2 min active rest HIIT B: 30 min of 4 × 4 min intervals at 80%–90% V̇O2peak with 3 min rest and 5 min active stage	8 weeks Cycle Ergometer 3x/week for 40 min MICT: 50%–60% V̇O2peak	Markers of cellular and circulating inflammation	HIIT and MICT protocols did not cause any immediate adverse negative effects on immunity. kidneyTx recipients. can exercise at a range of high intensities without worrying about long-term alterations to immune parameters.	None Reported	Cannot be sure that the difference seen for circulating immune cell subsets are the same at the tissue level. The use of muscle biopsies with the intention of immunostaining would have generated practical data adding to the validity of this study. Labeling cells in culture with a fluorescent marker such as carboxyfluorescein succinamidyl ester would have allowed information about individual cells. Assessing the proliferation and responsiveness of cells would add evidence to the interaction of exercise and immunosuppressive medications on immune cell subsets and may draw some conclusions on timing immunosuppressive medications around exercise.	Calcineurin Inhibitors Steroids
Masschelein et al. (34)	Belgium	To asses Mont Ventoux effects on patient-reported and perceived barriers and facilitators to physical activity	Heart Kidney Liver Lung	TxCYC (transplant cyclist) Age 47.9 ± 11.2 *n* = 47 7 Female 40 Male TxHIK (transplant hikers) Age 54.2 ± 11.5 *n* = 18 5 Female 13 Male	HCON (healthy control) Age 49.5 ± 11.0 *n* = 91 Female Male TxCon (transplant control) Age 50.1 ± 11.5 *n* = 213 37 Female 176 Male	More than 6 weeks TxCYC 3.9 (2.3–9.4) TxHIK 3.04 (2.3–7.4) TxCON 4.95 (2.4–8.9)	6 months Cycling and Hiking TxCYC Cycled 3x/week for 30 min per session 3 phases varying from 60 to 90% of individual peak HR (phase 3 HIIT) TxHIK Hiked 3x/week two short hikes for 30 min and one long for 2–8 h per session	6 months HCON Encouraged to train 3x/week TxCON Not stated	Physical activity, health-related quality of life, mental health and depressive symptomatology, anxiety and stress	A six-month dose of the MVT exercise training intervention lowered stress in the hiking transplant recipients, while other measurements remained stable. Patient reported barriers in participating transplant recipients were similar to those of their healthy counterparts but more favorable than those of the matched control transplant recipients. Control-group transplant recipients were less physically active and reported higher barriers, and lower facilitators to physical activity, and ranked these barriers and facilitators differently from the other three groups. This indicates that initiation and continuation of a physically active lifestyle in sedentary transplant recipients will require physical activity and exercise interventions tailored closely to their needs.	None reported	Selection bias as the least motivated patients were likely to leave the study prematurely Used self report report measures which can lead to social desirability bias Issue with international physical activity questionnaire terminology The intervention protocol differed between transplant recipients and HCON Did not exclude patients who did not complete the survey during one or more of their follow-up assessments, leading to increasing variability in the study sample.	

EG, exercise group; CG, control group; HIIT, high intensity interval training; TR, transplant recipient; BL, bilateral; SET, supervised exercise training; LV, left ventricular, Tx, transplantation; FMD, flow mediated dilation; HRQOL, health related quality of life; Vemax, maximal velocity; CAV, cardiac allograft vasculopathy; IVUS, intravascular ultrasound; a-v O2, arterio-venous oxygen; MICT, moderate intensity continuous training; PA, physical activity; HR, heart rate; BMD, bone mineral density; CON, continuous moderate exercise; BMI, body max index; SOTRs, solid organ transplant recipients; OCT, optical coherence tomography; MIT, maximum intima thickness; IMR, intima-media ratio; CVD, cardiovascular disease.

**Table 3:** Strenuous exercise.

Study/year	Country	Study objective	Organ Group	Exercise group (sample size/characteristics)	Control group s(sample size/characteristics)	Time post transplant	Description of sport/activity	Outcomes studied	Main findings	Adverse effects	Study limitations	Immunosuppressive therapy
Königsrainer et al. (14)	Germany	To analyse peripheral blood in healthy athletes and transplant recipients who participated n the Euregio cycling tour 2009 before and immediately after they performed 81 km of cycling	Kidney	*n* = 10 Age 43.5 ± 2.2 years All Male	*n* = 10 healthy Age 49.2 ± 7.2 years All Male	Not mentioned	Engaged in the Euregio cycling tour spanning 3 days, covering a total distance of over 300 km and ascents above 1,800 m in altitude.	Outcomes: neutrophils, gene expression	Relative increase of neutrophils n transplant recipients was significantly smaller than controls after exhausting exercise. The total number of circulating neutrophils after exhausting exercise similar in both groups. The blood neutrophil counts at rest, and also one day after exercise in TR were significantly higher than the counts in the controls. There was significantly higher expression of differentially up-regulated genes in controls compared with transplant recipients. Control athletes demonstrated a higher immune response regulation than the transplant recipients. Transplant recipients showed a significant activation of genes related to cell metabolism, but genes related to the immune response were missing. Most of the observed changes returned back to normal by the next day.	None Reported	Small sample size Only male	Tacrolimus Cyclosporine, Mycophenolate-Mofetil
K**ö**nigsrainer et al. (35)	Germany	To evaluate the effect of bacterial endotoxin contact after exhausting exercise in transplant recipients, who are innately immunosuppressed by their medication.	Kidney	Same as Königsrainer et al. (14)	Same as Königsrainer et al. (14)	Not mentioned	Same as Königsrainer et al. (14)	Outcome: gene Expression	Markedly oppositional pattern of gene expression in transplant recipients compared with controls after LPS incubation, directly after exhausting exercise. Immune response genes were significantly over-represented in controls immediately after the exhaustive exercise bout with LPS stimulation, whereas numerous apoptotic genes were over-represented in transplant recipients. Exhaustive exercise followed by pathogen contact, could lead to an increased risk of infection and cell damage in transplant recipients. The immune system in transplant recipients is impaired by the effect of exhausting exercise, pathogen contact and additionally by the immunosuppressive medication. This could lead to an increased risk of infection in transplant recipients with potential cell damage and its consequences after exhaustive exercise.	None Reported	LPS incubation of peripheral blood is not comparable to a real infection of an athlete Small sample size Only Males	Same as Königsrainer et al. (14)
Cappuccill et al. (36)	Italy	Evaluated the effect of a 130-km cycling race on inflammatory cytokines and adiponectin levels in transplant recipients.	Kidney Liver Heart	*n* = 19 transplanted cyclists Age 52.1 ± 9.0 All Male *n* = 32 Sedentary transplant recipients Age 54.5 ± 7.4 All Male.	*n* = 35 healthy amateur cyclists Age 50.0 ± 10.0 years All Male	TC (years) 9.3 ± 5.1 STR (years) 7.4 ± 6.6	Cycling race: 130 km path which included 4 hills with 1,871 meters of altitude drop.	Outcomes: circulating(IL-6, TNF-a, IFN-g, and adiponectin	Transplanted patients in good clinical condition, with a well-tolerated immunosuppressive therapy and proper training, can benefit from physical activity, even at a competitive level. Marathon cycling induced some changes in inflammation parameters that were transient and superimposable between physically active healthy and transplanted cyclists. The comparison with sedentary transplant recipients revealed a significant lowering in the circulating concentrations of certain proflogistic indexes as a possible effect of sporting activities on systemic inflammation.	None Reported	Small sample size Anti-inflammatory cytokines were not assayed Only Males	Tacrolimus Cyclosporine Steroids Mycophenolic acid Everolimus
Mosconi et al. (37)	Italy	To evaluate kidney function parameters in a group of TP in comparison with HV involved in a long-distance road cycling race: length 130 km and total uphill gradient, 1,871 m.	Kidney Liver Heart	*n* = 19 transplanted cyclists (TP) Age 52.1 ± 9.0	*n* = 35 healthy amateur cyclists (HV) Age 50.0 ± 10.0	(years) 9.3 ± 5.1	Cycling race: 130 km path which included 4 hills with 1,871 meters of altitude drop.	Renal function parameters: creatinine, eGFR, urea, uric acid, urine specific gravity, microalbuminuria, and proteinuria	Selected and well-trained organ-transplanted patients can perform an intensive bout of exercise, displaying temporary modifications on kidney function parameters comparable to healthy subjects.	None Reported	Small sample size Transplant patients were already active cyclist Only males	Tacrolimus Cyclosporine Steroids Mycophenolic acid Everolimus
Schrutka et al. (38)	Austria	To characterize the cardiopulmonary response to high altitude in lung transplant recipients.	Lung	*n* = 14 42 (25–65) 3 female 11 Male	*n* = 29 45 (30–61) 10 female 19 Males	(years) >1 year	Climbed the highest peak in North Africa (Mount Jebel Toubkal; 4,167 m)	Outcomes: Vital signs, repeated transthoracic echocardiography, functional capacity testing, pulmonary function tests, capillary blood sampling	Strenuous exercise in healthy lung transplant recipients is safe. However, the poorer cardiopulmonary performance in the 6-minute walk test and the lack of right ventricular cardiac adaptation may indicate underlying autonomic dysregulation.	No severe complications were observed	Logistic constraints impeded a uniform assessment over the entire course of the trekking. Full echocardiographic assessments of participants would have significantly improved understanding of cardiac adaptation at high altitudes. Cold temperature, short sleeping times, and unfavorable logistic possibilities. Unequal number of participants per study group VO2 max and heart rate variability measurements could have provided more evidence of the autonomic activation in the study participants. Echocardiographic estimation of TAPSE and sPAP has methodological limitations.	Not Reported
Pirenne et al. (39)	Belgium	To compare the liver transplant patients and normal healthy subjects in terms of; capacity to perform intense physical activities, tolerance to altitude and hypoxia, and susceptibility to acute mountain sickness.	Liver	*n* = 6 Age: 39 ± 5 3 Female 3 Male	*n* = 15 Age: 42 ± 5 3 Female 12 Male	(years) 3.8 (range: 2–5)	Participated in a trek up Mount Kilimanjaro (5,895 m), Tanzania.	Physical performance Acute mountain sickness	Selected and well-prepared liver transplant recipients can perform strenuous physical activities and tolerate exposure to high altitude similar to normal healthy people. 83% of transplant subjects and 84.6% of control subjects reached the summit.	One control and one transplant subject abandoned the study due to gastroenteritis in the former and physical exhaustion and lower oxygen saturation in the latter.	Small sample size Transplant and control subjects received different preventions for acute mountain sickness and caution should be used when comparing these two groups. Dexamethasone administration was limited to the summit bid and were doses lower than those usually recommended were given, which might explain the trend in lower acute mountain sickness scores in transplant subjects during the last ascent compared with acetazolamide-treated controls	Tacrolimus

TR, transplant recipients; LPS, lipopolysaccharide; IL-6, interleukin-6; TNF-a, tumor necrosis factor alpha; IFN-g, interferon gamma; HV, healthy volunteers; eGFR, estimated glomerular filtration rate.

**Table 4:** Case studies.

Author/year	Country	Organ Group	Participants Characteristics	Time post transplant	Comorbidities	Exercise/training regimen	Competitive performance	Achievements	Physiological measures	Adverse effects	Immunosuppressive therapy
De Smet et al. (40)	USA	Heart	48 year old male Active pre transplant playing tennis, soccer, skiing	9 months		Walking and bicycle ergometer for 30 min a day	20 km jogging competition	Completed in 2.5 h (12,031 of 16,000 competitors)	Heart rate stabilized between 150 and 160 Resting LVEF was 46% Blood pressure 130/80mmHG during recovery High blood lactate	Muscular cramps in calves	Cyclosporine, Prednisone
Fink et al. (74)	USA	Heart-Lung	28 year old male	10 years	CF Pulmonary disease, Acute severe rejection, Acute pulmonary hemorrhage, Pseudomonas infections, Transient renal failure, CF liver disease, Diabetes mellitus.	Strenuous training of running and swimming	Participated in sporting events for transplant patients.	Won many competitions.	Supernormal aerobic and ventilatory capacity High oxygen uptake (128% of predicted), oxygen pulse (174% of predicted), Delayed onset of ventilatory anaerobic threshold (2,290 vs 1,840 ml/min)		Steroids, Cyclosporine
Browne et al. (41)	UK	Kidney	47 years old	14 months		Lifting heavy flat pack furniture and Intense gym workouts days prior to his symptoms			haematoma over the lateral aspect of the transplant kidney with displacement of the hilum anteriorly and medially. Vascular supply to the kidneys was compressed.	Anuria Dull aching pain over transplant kidney Unable to confirm whether the exercise was the main cause of the hematoma	Tacrolimus, prednisolone, azathioprine
Howard Jones (16)	UK	Kidney	58 year old female Swam and played hockey prior competitively as a teen Competes in Masters swimming.	2 years	Renal anemia	Cardio and light weights. Swimming 3 times per week in 1 h sessions completing 2,500 m		British Games- 2 gold medals, 3 in the World games in Kobe, 5 in france, 5 in canada. Holds transplant games record in 5 events. Competed in great Britain masters championships. Gold, silver and bronze in major national and European championship. Was ranked 5th in the world			Cyclosporine, prednisone
Haykowsky et al. (42)	Canada	Heart	45 year old male Was active before transplantation performing aerobic exercise 2 to 3 days per week.	18 years	Hypertension	Participated in a 12-week exercise study	Half-ironman triathlon Olympic distance triathlon	Completed in 6 h and 15.9 min (416th of 611 competitors) Completed in 3 h and 2.1 min (75th of 126 competitors)	VO2peak of 59 ml/kg/min Rapid increase in HR from rest to peak exercise (change in HR of 81 bpm) 177 peak HR during exercise study	Not Reported	Calcium channel blockers
Haykowsky et al. (43)	Canada	Heart	54 year old male	27.7 years	Not Reported	Not Reported	2013 and 2014 Boston Marathon	Completed in 4 h 33 min and 54 s finished 24,032 out of 31,931 runners (ranked in the top 75% of all finishers)	VO2peak of 49.5 ml/kg/min Mild left ventricular dilation Preserved systolic function Supranormal left ventricular diastolic function Average HR during 2014 race (148 beats/min)	None Reported	Not Reported
Haykowsky et al. (44)	Canada	Heart	24 year old Male Professional cyclist with a high VO2peak prior to transplantation (71–72 ml.kg.min)	14 months	Not reported	Resumed training regimen typical for a competitive cyclist including high-volume and high intensity protocols	Professional cycling		Vo2peak 64 ml·kg–1 min–1 post transplant maximal HR fluctuated between 169 and 179 bpm, resting HR decreased by 10 to 22 bpm, and 2-min recovery HR increased by 16 bpm.	Not reported	Not Reported
Haykowsky et al. (73)	Canada	Heart	42 year old male Completed 6 Ironman distance races prior to transplantation	28 months	Not Reported	Not reported	Ironman Triathlon	first heart transplant recipient to finish the Ironman World Championship (race time 12 h and 30 min, 1,490th place out of 1,985 official finishers). Also finished the Ironman European Championship event Race time (11 h and 39 min (1,282th place out of 2,396 official finishers). Fastest Ironman finish times ever reported for a heart transplant recipient and the first report of a heart transplant recipient completing 2 Ironman races within 14 weeks. Competed in >60 endurance races	Resting ejection fraction of 79% V02max of 53 ml/kg/min (92% of age-predicted value for an endurance-trained individual	None Reported	Not Reported
Einollahi et al. (45)	Iran	Kidney	29 year old male High level athlete prior to transplantation	2.7 years	Hyperlipidemia	Bodybuilding and other vigorous exercises	Professional Boxing			Not Reported	Cyclosporine, Prednisone, Mycophenolate mofetil

LVEF, left ventricular ejection fraction; CF, cystic fibrosis; HR, heart rate; Bpm, beats per minute.

### 3.1 Overview of the studies offering high intensity exercise

#### 3.1.1 Vo2peak

When comparing HIIT against moderate intensity continuous exercise (MICT) protocols, or standard care, HeartTx (Tx = transplantation) recipients have demonstrated a significantly greater mean improvement in VO2peak throughout all studies with HIIT (HIIT; + 4.17 ml/kg/min, Control; + 2.07 ml/kg/min,) (19, 20, 23, 28, 46). However, high baseline VO2peak values in Nytroen et al. 2012 may reflect bias in the population chosen (20). A 1-year follow up of participants from Nytroen et al. 2012 found the favorable increases in VO2peak within the HIIT group persisted, (HIIT; + 3.2 ml/kg/min, Control; 0 ml/kg/min), with a statistically significant between group difference (21). Although, 5-years later the HIIT abstinence resulted in a loss of the improvement in VO2peak, suggesting the importance of maintaining a HIIT exercise routine (22). A 3-year follow up of Nytroen et al. (23) which compared HIIT to MICT demonstrated that the HIIT group kept their superior effects compared to the MICT group, but this difference was not significant between groups (24). In LungTx recipients, HIIT increased VO2peak but this was not a significant improvement compared to the standard care group. This may be attributed to the little adherence to the exercise protocol within the HIIT group (26). These benefits appear to be the greatest if the intervention is undertaken within 2 years after Tx (26). This highlights the importance of implementing interventions aimed at preserving, or improving aerobic fitness early in the post-transplant period, which may consequently decrease coronary heart disease risk. In a single armed intervention including LungTx, HeartTx, KidneyTx, and LiverTx recipients, HIIT showed a significant overall increase in VO2peak (+3.2 ml/kg/min), with the greatest increase in HeartTx recipients (+4.6 ml/kg/min) (30). There was no significant increase in VO2peak within LiverTx recipients which could be due to their high baseline values and their steroid free regimen (30). Billany et al. on the other hand found a greater increase with MICT (+3.72 ml/kg/min) compared to HIIT A (+2.78 ml/kg/min) and HIIT B (+2.83 ml/kg/min) in KidneyTx recipients (32). However, this may be attributed to their higher-than-normal baseline VO2peak values compared to HIIT A and HIIT B (HIIT A: 30 min of 4-, 2-, and 1-min intervals; 80%–90% watts at V̇O2peak with 2 min active rest; HIIT B: 30 min of 4 × 4 min intervals at 80%–90% V̇O2peak with 3 min rest and 5 min active stage). While HIIT holds promise for improving cardiovascular health markers such as VO2peak in SOTRs, particularly early post-transplant, there is a need to explore long-term adherence and the effects of diverse high intensity exercise protocols across different transplant types. Additionally, determining optimal training protocols remains an area necessitating further investigation.

#### 3.1.2 Endothelial function and inflammatory biomarkers

In HeartTx recipients, cardiac allograft vasculopathy emerges as a progressive atherosclerotic manifestation (47). Unlike typical atherosclerosis, cardiac allograft vasculopathy distinguishes itself by the thickening of the inner arterial wall, and a widespread constriction of smaller blood vessels leading to elevated rates of health complications and mortality (48). Studies have shown physical activity to offer atheroprotective effects, with HIIT demonstrating even greater benefits in populations with coronary artery disease (20, 47). This is by way of reduction in chronic inflammation which is associated with endothelial dysfunction, and increased formation and progression of atherosclerotic plaques (49). An 8-week HIIT intervention in HeartTx recipients significantly increased flow mediated dilation of the brachial artery compared to sedentary individuals, (HIIT; + 3.1%, Control; + 0.3%) with a statistically significant difference between control and intervention groups (19). The patients were although treated with vasodilators which could interfere with the effect of exercise on flow mediated dilation making it more challenging to draw concrete conclusions (19). Likewise, 12 weeks of high intensity supervised exercise training did not have an impact on brachial artery endothelial-dependent, or -independent vasodilation compared to no training (46). Interestingly, when compared to MICT, HIIT does not seem to elicit significantly greater improvements in flow mediated dilation, arterial stiffness, and endothelial function (23, 28). Modulation of low-grade inflammation in HeartTx recipients by exercise training could have a positive impact on endothelial function, and potentially, long-term outcomes (19). Yet, the superior effects of HIIT compared to MICT or usual care on the reduction of inflammatory biomarkers yields mixed results. In comparison to MICT, HIIT shows no significant effect on inflammatory activity including, tumor necrosis factor-alpha, orosomusciod, interleukin 6, and interleukin 10, or adiponectin levels in HeartTx or KidneyTx recipients (29, 33, 50). However, when compared to sedentary controls, HIIT resulted in a significantly greater mean reduction in inflammatory responses in HeartTx recipients (19). These included, high-sensitivity c-reactive protein (HIIT; −0.60 mg/ml, Control; −0.27 mg/ml) and pro-atrial natriuretic peptide (HIIT; −73.2 nmol/ml, Control; −12.1 nmol/ml) (19). Using intravascular ultrasound analysis 3 months post innervation, Nyotrean et al. found HIIT among maintenance HeartTx recipients resulted in a significantly slowed rate of cardiac allograft vasculopathy progression compared to usual care (50). This was evident by a significantly smaller mean increase the percent atheroma volume (PAV) (HIIT; + 0.9%, Control; + 2.5%), and total atheroma volume (TAV) (HIIT; 0.3 mm^3^/mm, Control; 1.1 mm^3^/mm) with significant differences between the groups (50). It should be noted that baseline intravascular ultrasound parameters were numerically higher in the control group at baseline suggesting population homogeneity was not met (50). A 3-year follow-up showed mild cardiac allograft vasculopathy in the HIIT group remained stable (5 n), while the control group saw a decrease (8 to 7 n). Similar results were evident when HIIT was compared to usual care during a 5-year follow-up (22, 24). The effects were lost in parallel to decline in VO2peak suggesting a potential need for exercise maintenance (22). However, conflicting evidence exists. A 6-month intervention in HeartTx recipients comparing HIIT to MICT found a greater cardiac allograft vasculopathy prevalence following exercise, increasing by 50% in both groups at follow up (HIIT; 4–8 n, MICT; 6–12 n) (31). These findings may potentially be explained by the usage of coherence tomography which produces images with 10 times the spatial resolution of intravascular ultrasound, providing a clear delineation of the arterial vessel and *in vivo* vessel histological analysis (51). Additionally, while physical activity offers well-documented cardiovascular benefits, these advantages may follow a curvilinear dose-response relationship. Engaging in exercise beyond an “optimal dose” in terms of duration and intensity could potentially increase the risk of coronary plaque development, but this remains debated with inconclusive data (52, 53). Given the elevated cardiovascular risk in SOTRs, stricter pre-participation screening, including cardiopulmonary exercise testing, functional imaging, and potentially coherence tomography scans may be warranted before engaging in vigorous exercise (54). Furthermore, the atheroprotective benefits of HIIT and MICT seem to coincide, while HIIT only shows superior effects compared to usual care or sedentary protocols.

#### 3.1.3 Blood pressure

Hypertension and its clinical complications are common amongst SOTRs with a prevalence of 50%–80% (55). In healthy subjects, regular cardiovascular exercise has been shown to lower resting blood pressure. The heart is strengthened allowing for a greater volume of blood to be pumped with less effort (17). A HITT intervention compared to sedentary controls in HeartTx recipients significantly reduced resting systolic blood pressure (HIIT; −15 mmHg, Control; + 1 mmHg) (19). No significant changes were evident in either group for resting diastolic blood pressure (19). When compared to MICT, HIIT again induced a greater reduction in systolic blood pressure in HeartTx recipients, but these differences were again not statistically significant (28). Likewise, no changes in diastolic blood pressure were evident (28). However, these studies should be interpreted with caution as they were not powered to detect changes in blood pressure (19, 28). In KidneyTx recipients both MICT and HIIT interventions led to an overall decrease in systolic blood pressure (−6 mmHg) with the greatest decrease in MICT (−10 mmHg) (32). Unlike the previous studies diastolic blood pressure also decreased overall (−5 mmHg) with the greatest decrease in MICT (−8 mmHg). This may be explained by group heterogeneity as the MICT group had higher baseline values (32). It should be noted that hypertensive individuals should take precaution when engaging in high intensity exercises as it induces a sudden spike in blood pressure (8). The combination of high blood pressure and the intense stress of HIIT can increase the risk of cardiovascular events such as myocardial infarctions and strokes (11). Hypertensive SOTRs should do their due diligence in regular blood pressure monitoring and talk to their physician regarding potential medication alteration before engaging in HIIT.

#### 3.1.4 Cardiopulmonary performance variables

An improvement in cardiopulmonary variables through exercise is crucial for SOTRs due to its significant implications for cardiovascular health. Enhanced heart rate response, increased cardiac output, and improved systolic function are indicators of improved cardiovascular fitness, which can mitigate the risk of cardiovascular complications post-transplantation (56). Furthermore, these responses are often blunted in SOTRs, especially in HeartTx recipients (56). For individuals aspiring to compete recreationally or professionally, optimizing these variables through exercise interventions becomes not just desirable, but imperative. Dall et al. reported a greater increase in heart rate peak with HIIT (+4.3 bpm), contrasting with MICT (+1.2 bpm) in HeartTx recipients (28). Additionally, HIIT exhibited a significant decrease in resting heart rate with no change in the MICT exercise group, and no significant between group differences (28). Heart rate reserve had a marginal increase with HIIT (+5.3 bpm), but not with MICT (+0.7 bpm) while showing a significant between group difference (28). When compared to MICT, HIIT did not result in significantly greater improvements in cardiac output, VE/VCO2slope, ejection fraction, left ventricular end-diastolic diameter, heart rate max, peak heart rate, heart rate reserve, and left ventricular end-systolic diameter (23). These results remained consistent during a 3-year follow-up (22)**.** However, in comparison to usual care, HIIT resulted in a significant increase in peak heart rate (+4 bpm) with no change in the control group (0 bpm), and a significant in between group difference 1 year post intervention in HeartTx recipients (21). Moreover, HIIT improved left ventricular movement but did not have a major impact on diastolic function (21). This suggest that while HIIT might improve some aspects of left ventricular function in HeartTx, it likely does not directly enhance the heart muscles contractility or significantly increase the amount of blood pumped per beat (24). Haywosky et al. found during rest and exercise, HIIT did not result in significant changes in cycle exercise end-diastolic cavity area, end-systolic cavity area, stroke area and area ejection fraction after 12 weeks of supervised exercise training or no training in HeartTx recipients (46). In KidneyTx recipients both HIIT and MICT were able to decrease resting heart rate in all interventions by (−5 bpm) with the greatest decrease in HIIT B (−11 bpm) (32). Stroke volume had an overall increase (+3.59 ml/beat) in all interventions with HIIT B having the highest increase (+3.77 ml/beat) (32). Cardiac output had an overall decrease (+0.52 ml/beat) but only significantly increased in HIIT A (+0.07) (32). Moreover, total peripheral resistance decreased overall (−110.74 dyn s cm^5^), with the greatest decrease in MICT (−37.08 dyn s cm^5^) (32). In an intervention comparing all transplant groups, peak heart rate, and VE/VCO2 slope did not change within any of the groups, while resting heart rate did decrease significantly (−4.7 bpm) (30). LungTx and KidneyTx had the greatest drop in resting heart rate (−5.9 bpm and −4.4 bpm, respectively) (30). HIIT appears to be a promising strategy for improving some aspects of cardiovascular health in SOTRs. Literature shows significant increases in peak heart rate, and heart rate reserve following HIIT compared to controls in SOTRs. Additionally, HITT may improve left ventricular annular systolic displacement, indicating better overall heart movement. However, the impact on other key markers seems less consistent.

#### 3.1.5 Physical activity and sedentary time

A significant proportion of SOTRs struggle to meet recommended physical activity levels (9), and HIIT may present a potential solution. HIIT is time efficient and has demonstrated greater enjoyability compared to traditional exercise throughout the general active population (57). This could lead to increased adherence and reduced sedentary time which remains a problem in the SOT population (58). However, existing research on the impact of HIIT on daily physical activity in SOTRs paints a complex picture. Follow-up studies 1- and 5-years post HIIT in HeartTx recipients demonstrated that most of the participants only engaged in moderate-intensity daily activity levels (30–60 min) (21, 22, 24). Similarly, Ulvestad et al. found no significant difference in physical activity or sedentary time among LungTx recipients following HIIT or usual care (32). Likewise, in KidneyTx both HIIT and MICT led to an increase in physical activity levels post intervention but without significant between group differences (32). A cycling and hiking intervention consisting of HeartTx, LungTx, KidneyTx, and LiverTx recipients found the continuation of a physically active lifestyle in sedentary transplant recipients will require physical activity and exercise interventions tailored closely to their needs (34). The intervention led to an increase in physical activity at follow-up but the results should be interpreted with caution as social desirability bias might have inflated self-reported data in some studies (24, 34). The current evidence regarding the effect of HIIT on daily physical activity and sedentary time in SOTRs is inconclusive. While HIIT holds promise for promoting activity in this population, retention of HIIT seems to be minimal. Studies provide supervision and group cohesivity which can bolster motivation. Further research needs to focus on how to translate these factors post interventions to address adherence challenges.

#### 3.1.6 Body composition

Post-SOT patients are often prescribed corticosteroid medication and calcineurin inhibitors which are associated with muscle atrophy and osteoporosis (59). This is most prevalent after LungTx as they have a 12%–15% bone mineral density reduction within the first 2 months (60). This entails a higher incidence of fractures at four to five times compared to the general population (61, 62). Likewise, by 12 months post-transplant, there is a trend indicating higher proportion of overweight or obese individuals among transplant recipients as categorized by the World Health Organization guidelines: kidney (53.4%), liver (51.5%), heart (51.7%), and lung (33.1%) (63). HIIT combined with strength training in LungTx and HeartTx recipients led to increased trabecular bone score of the lumbar spine density, increased total body mass and leg lean tissue mass, and decreased subcutaneous fat compared to usual care (27, 46). However, other body composition measures such as lean body mass, bone mineral density, and fat body mass did not differ significantly from standard care (27, 46). This was coupled with greater increases in the HIIT group's leg press, quadriceps, hamstrings, and chest press maximal strength. However, there were no significant differences between groups or changes in arm curl or latissimus dorsi pulldown (20, 27, 46). In various transplant groups (heart, lung, kidney, liver) HIIT significantly decreased body weight (−1.1 kg) and BMI (−0.4 kg/m^2^), while increasing Wmax (maximal work capacity) in all groups (+12.8 W) (30). Nytroen et al. 2019 found no significant differences in any body composition measures between HIIT and MICT in HeartTx recipients, with these results persisting over several years (22, 23). Moreover, HIIT participants showed a significantly higher mean change in muscular exercise capacity (muscles ability to generate force and sustain effort over a period of time) 1 year post intervention, but these results were not significant 5 years later (21, 22, 24). Body composition and HIIT in KidneyTx recipients showed contradicting results, with one study finding increased body fat (+0.83%), BMI (+0.10 kg/m^2^), and decreased lean muscle mass (−0.45 kg) after both HIIT and MICT, highlighting potential exercise intensity-dependent effects (32). While HIIT shows promise in potentially improving bone health and muscle mass in some SOTR, the results across studies are inconsistent. Regular screening practices and bone mineral density measurements should be undertaken in SOTRs wishing to participate in high intensity endeavors that require high impact.

#### 3.1.7 Health-related quality of life

In SOTRs, a higher perceived health related quality of life (an individual's perceived physical and mental health over time) has been associated with greater motivation to adhere to medication schedules, follow-up appointments, and healthy lifestyle changes (64). This can lead to better long-term graft survival and overall health (64). HIIT interventions have demonstrated significant improvements in physical and mental health, and overall health related quality of life (small-to-moderate effect size) in clinical and non-clinical populations (65). Notably HIIT appears to be equally effective as MICT in enhancing health related quality of life, offering a time-efficient alternative in the general population (65). A 12-month intervention in HeartTx recipients comparing HIIT and usual care found no significant changes in health-related quality of life (20). However, a 9-month exercise protocol in HeartTx recipients showed both HIIT and MICT groups increased anxiety but decreased depression, but these results were not significantly different between groups (23). Likewise, there was a significant increase in PCS scores within both groups, but this was not significantly different between groups (23). The same participants 3 years later significantly increased physical functioning and role scores (25). Vitality only significantly increased in MICT group, while only the HIIT group significantly increased in role emotional with a significant in between group difference (25). Compared to usual care, 5 years post a HIIT intervention, HIIT had higher scores in all variables as assessed by the SF-36 version 2 but only the role physical score was significantly higher (24). All patients showed higher depression rates from baseline to 5-year follow-up with but with no between group differences. Anxiety decreased in the HIIT group (−1%) and increased in the control (+9%) group but these differences were not significant (24). LungTx recipients undergoing HIIT scored significantly greater on the SF-36 MCS compared to usual care (32)**.** Both HIIT and continuous moderate exercise led to significant improvements in physical function, energy and general health, while reducing markers of anxiety and depression (32). None of these variables showed between group differences and had a significant loss of their positive responses during the washout period (29). In various transplant groups (lung, heart, kidney, liver) participating in hiking and cycling, the exercises initially led to significantly improved health-related quality of life and mental health scores compared to the transplant control group. However, the benefits were not sustained in the long term (25). Additionally, seasonal variations appeared to influence the results, with some improvements observed before or after the summer months. Despite some transient improvements, stress levels remained largely unchanged across all groups (25). Long-term follow-up studies present mixed findings: some show sustained improvements in specific health related quality of life domains like physical functioning and role emotional, while others observe no significant differences between HIIT and control groups. Most of these studies although had missing participants at follow-up and lacked disease specific HRQOL questionnaires which could have been more sensitive in detecting differences between groups.

### 3.2 Overview of the studies including strenuous sports and exercises

#### 3.2.1 Immunological response

Research conducted in healthy populations has demonstrated that prolonged exercise exceeding 60% of VO2peak can induce transient immunosuppression (66). These consequences are further amplified for SOTRs who can experience slower recovery time from infections, potentially leading to serious illnesses, or worse, organ rejection due to their already immunosuppressed state (67). This is by way of reduction in leukocyte function and alteration in inflammatory parameters (68). KidneyTx recipients undergoing a cycling tour spanning over 300 km revealed a small relative increase in neutrophils compared to controls with a majority of cell activation being found in cell metabolism while genes related to immune function were not expressed (68). When bacterial endotoxin was introduced to their blood sample after exhausting exercise, 86 up-regulated and 4 down-regulated genes were detected in transplant recipients, while 151 up regulated and 18 differentiated downregulation genes were detected in controls (35). Immune response genes were immediately overrepresented in the control group and the KidneyTx recipients had an overrepresentation of apoptotic genes (35). The immune system in SOTR seems to be impaired by the effect of exhausting exercise, pathogen contact and additionally by the immunosuppressive medication (35). This could lead to an increased risk of infection in transplant recipients with potential cell damage and its consequences after exhaustive exercise (35). More research is needed for a definite conclusion, but if strenuous exercise does further transiently suppress SOTRs immune function, a reduction in medication dosage before engaging in the strenuous activity should be evaluated.

#### 3.2.2 Inflammation and kidney function

A 130 km cycling race in KidneyTx, LungTx, and HeartTx recipients found marathon cycling induced similar inflammation effects compared to healthy individuals (36). When compared to sedentary SOTRs, they had lowered circulating concentration of certain proflogisitc indexes, indicating reduced systemic inflammation (36). Data regarding the functional kidney adaptations that occur during intense and prolonged physical exercise in SOTRs receiving immunosuppressive therapy with nephrotoxic effects remains understudied (69). Mosconi et al. found SOTRs displayed similar positive trends in renal function blood values compared to healthy controls except for urine specific gravity (37). Kidney function seemed to fail in concentrating urine at the peak intensity of the race, and this difference was again more evident 18–24 h after. This was attributed to possible tubular impairment, but overall, it was determined that SOTRs with no cardiovascular contraindications can safely participate in a long-distance road cycling race without acute signs of kidney damage (37).

#### 3.2.3 Strenuous altitude ascension

LungTx and LiverTx recipients have successfully summited high-altitude peaks such as Kilimanjaro (Tanzania) and Jebel Toubkal (Morocco) (38, 39). At their peaks at 5,895 m and 4,167 m above sea level, acute mountain sickness had a high prevalence rate among as ascenders at around 80% (38, 39). Functional pulmonary tests, echocardiographic and vital parameters all displayed similar results to healthy controls which is a favourable indication. However, LungTx recipients displayed poorer cardiopulmonary performance in the 6-minute walk test and the lack of right ventricular cardiac adaptation may indicate underlying autonomic dysregulation (38). It also should be noted that high altitude exposure could have a negative impact on the kidneys as hypoxia may trigger the development of acute and chronic kidney failure (70).

### 3.3 Individual reports documenting post-SOT performance at highly competitive or physiological levels

Rare instances stand out as noteworthy exceptions to the prevailing observations regarding comprised exercise capacity among individuals’ post-SOT (71). Alonzo Mourning and Sean Elliot, returned to play in the NBA after a kidneyTx, and Howard Jones won gold, silver and bronze at the European aquatic games after a kidneyTx as well (16). This showcases the potential for remarkable athletic achievements post SOT. A retrospective analysis of the retrieved case studies reveals intriguing insights into the outcomes and challenges encountered by SOTRs across various sporting disciplines, and post-transplant periods. Notable findings include instances of enhanced aerobic and ventilatory capacities among athletes, as evidenced by supernormal oxygen uptake and delayed onset of ventilatory aerobic threshold (18, 42–44, 72). However, these favorable physiological adaptations may be accompanied by challenges such as muscular cramps, hematoma formation, and altered renal function, potentially attributable to immunosuppressive therapies and pre-existing comorbidities (40, 41). Despite these hurdles, several transplant recipients have demonstrated remarkable achievements in competitive sports, ranging from marathons and triathlons, cycling, swimming and bodybuilding, underscoring the potential for post-transplant individuals to engage in high-level athletic pursuits. Of particular interest are the cases of athletes achieving notable milestones, such as completing Ironman distance races spanning over 12 h and professional boxing competitions (45, 73). A major caveat throughout all of these cases is that the accomplishments were attained by individuals who were physically active, or already competing professionally pre-transplantation. Such physiological recuperations are therefore not expected to be attained by recipients who were inactive pre-transplantation. They are indicative of the exceptional dedication, resilience, and pre-existing athletic capacity possessed by the individuals.

### 3.4 Safety

None of the identified studies reported any adverse effects following exercise, suggesting high intensity and strenuous exercise is generally well-tolerated and safe for stable SOTRs. This however should be interpreted carefully as all the studies lacked *a priori* definition of adverse effects.

## 4 Limitations

None of the high intensity interventions or cross-sectional strenuous activity studies found adverse effects post exercise. However, Styelmans et al. hypothesized that most studies relied on retrospective self-reporting of adverse events. This approach may have led to the omission of harms that patients considered insufficiently severe or significant, as well as those they did not perceive as connected to the study (7). They further state that not only are the safety parameters poorly defined and described, but the authors also fail to inform the reader whether dropouts may have been related to adverse events (7). Future studies should implement a priori definitions for adverse events and include a prospective evaluation of potential harms. Additionally, researchers should clearly describe whether dropouts could potentially be related to the applied intervention. Furthermore, some studies lacked active control groups, were underpowered, and had insufficient durations. They also included heterogeneous populations and showed low adherence to protocols. Many studies were not powered to detect secondary outcomes and exhibited bias in selection criteria, as only stable, healthy SOTRs were chosen. These factors make it challenging to generalize the findings to the entire transplant population. Additionally, some participants were not able to attain the required power output demands, and many follow up studies had participant attrition. Lastly, social desirability bias might have led to skewed responses in self-reports, especially for physical activity levels.

## 5 Conclusion

The existing body of literature suggests high-intensity exercise presents a viable avenue for safely enhancing various physiological parameters while reducing coronary heart disease prevalence. It is also able to elicit greater than, or equal benefits compared to moderate intensity exercise. Individuals who were active pre-transplantation are able to attain physiological measures equivalent to their healthy counterparts, and in rare instances surpass their pre-transplant thresholds. Furthermore, SOTRs were able to complete strenuous exercises with no reported adverse effects. However, in high intensity exercises, follow-ups indicate low retention rates for HIIT, which resulted in a loss in adaptations. Both strenuous exercises and HIIT interventions had no priori definitions of adverse effects. Future studies should therefore not only address methodological limitations, but also explore strategies to enhance the long- term high intensity exercise adherence. This would provide a more comprehensive understanding of HIIT’s efficacy and sustainability as a therapeutic exercise approach in clinical settings.

## References

[1] Wiltshire G Clarke NJ Phoenix C Bescoby C. Organ transplant recipients’ experiences of physical activity: health, self-care, and transliminality. Qual Health Res. (2021) 31(2):385–98. 10.1177/1049732320967915 33124516 PMC7750649

[2] Miller WL. Cardiovascular toxicities of immunosuppressive agents. Am J Transplant. (2002) 2(9):807–18. 10.1034/j.1600-6143.2002.20902.x 12392286

[3] Williams TJ McKenna MJ. Exercise limitation following transplantation. Compr Physiol. (2012) 2:1937–79. 10.1002/cphy.c110021 23723030

[4] Corbett C Armstrong MJ Parker R Webb K Neuberger JM. Mental health disorders and solid-organ transplant recipients. Transplantation. (2013) 96(7):593–600. 10.1097/TP.0b013e31829584e0 23743726

[5] Chekroud SR Gueorguieva R Zheutlin AB Paulus MP Krumholz HM Krystal JH Association between physical exercise and mental health in 1.2 million individuals in the USA between 2011 and 2015: a cross-sectional study. Lancet Psychiatry. (2018) 5(9):739–46. 10.1016/S2215-0366(18)30227-X 30099000

[6] Benedetti MG Furlini G Zati A Letizia Mauro G. The effectiveness of physical exercise on bone density in osteoporotic patients. Biomed Res Int. (2018) 2018:4840531. 10.1155/2018/4840531 30671455 PMC6323511

[7] Stylemans D Vandecruys M Leunis S Engelborghs S Gargioli D Monbaliu D Physical exercise after solid organ transplantation: a cautionary tale. Transpl Int. (2024) 37:12448. 10.3389/ti.2024.12448 38414660 PMC10898592

[8] Bednarczyk C Tansey CM Fontaine S Baker S Laberge É Mathur S Community-based exercise program for solid organ transplant recipients: views of exercise professionals and patients. Mcgill J Med. (2021) 19(1):218. 10.26443/mjm.v19i1.218

[9] Coates AM Joyner MJ Little JP Jones AM Gibala MJ. A perspective on high-intensity interval training for performance and health. Sports Med. (2023) 53(Suppl 1):85–96. 10.1007/s40279-023-01938-6 37804419 PMC10721680

[10] Villelabeitia-Jaureguizar K Vicente-Campos D Senen AB Jiménez VH Garrido- Lestache MEB Chicharro JL. Effects of high-intensity interval versus continuous exercise training on post-exercise heart rate recovery in coronary heart-disease patients. Int J Cardiol. (2017) 244:17–23. 10.1016/j.ijcard.2017.06.067 28648356

[11] Rojhani-Shirazi Z Abolahrari-Shirazi S Kojuri J Bagheri Z. Efficacy of combined endurance-resistance training versus endurance training in patients with heart failure after percutaneous coronary intervention: a randomized controlled trial. J Res Med Sci. (2018) 23:12. 10.4103/jrms.JRMS_743_17 29531564 PMC5842444

[12] Oliveira Carvalho V Guimarães GV Vieira ML Catai AM Oliveira-Carvalho V Ayub- Ferreira SM Determinants of peak VO2 in heart transplant recipients. Rev Bras Cir Cardiovasc. (2015) 30(1):9–15. 10.5935/1678-9741.20140055 Erratum in: Rev Bras Cir Cardiovasc. 2015 Mar-Apr;30(2):287. Campos-Vieira, Marcelo Luiz [corrected to Vieira, Marcelo Luiz Campos]. 25859862 PMC4389529

[13] Pederson BK Hoffman-Goetz L. Exericse and the immune system—regulation, integration, and adaptation. Physiol Rev. (2000) 80(3):1055–81. 10.1152/physrev.2000.80.3.1055 10893431

[14] Königsrainer I Zieker D Löffler M Bühler S Walter M Beckert S Influence of exhaustive exercise on the immune system in solid organ transplant recipients. Exerc Immunol Rev. (2010) 16:184–93. 20839499

[15] D'Ambrosio A Toulouse C Bélanger-Marceau S Savary S Mathur S Segatto B Characteristics and motivation of solid organ transplant recipients attending the Canadian transplant games. Transplant Proc. (2021) 53(2):581–9. 10.1016/j.transproceed.2020.06.041 33004224

[16] Howard-Jones J. A transplant athlete’s perspective. Ann Transplant. (2005) 10(4):57–8. 17037093

[17] American College of Sport Medicine. ACSM’s Guidelines for Exercise Testing and Prescription. Amsterdam, Netherlands: Wolters Kluwer (2018). Available online at: https://www.acsm.org/education-resources/books/guidelines-exercise-testing-prescription (cited 2024-05-26).

[18] Haykowsky MJ Riess K Burton I Jones L Tymchak W. Heart transplant recipient completes ironman triathlon 22 years after surgery. J Heart Lung Transplant. (2009) 28:415. 10.1016/j.healun.2008.12.024 19332274

[19] Hermann TS Dall CH Christensen SB Goetze JP Prescott E Gustafsson F. Effect of high-intensity exercise on peak oxygen uptake and endothelial function in long-term heart transplant recipients. Am J Transplant. (2011) 11:536–41. 10.1111/j.1600-6143.2010.03403.x 21219582

[20] Nytrøen K Rustad LA Aukrust P Ueland T Hallén J Holm I High-intensity interval training improves peak oxygen uptake and muscular exercise capacity in heart transplant recipients. Am J Transplant. (2012) 12:3134–42. 10.1111/j.1600-6143.2012.04221.x 22900793

[21] Rustad LA Nytrøen K Amundsen BH Gullestad L Aakhus S. One year of high-intensity interval training improves exercise capacity, but not left ventricular function in stable heart transplant recipients: a randomized controlled trial. Eur J Prev Cardiol. (2014) 21(2):181–91. 10.1177/2047487312469477 23185084

[22] Yardley M Gullestad L Bendz B Bjørkelund E Rolid K Arora S Long-term effects of high-intensity interval training in heart transplant recipients: a 5-year follow-up study of a randomized controlled trial. Clin Transplant. (2017) 31(1). 10.1111/ctr.12868 27865004

[23] Nytrøen K Rolid K Andreassen AK Yardley M Gude E Dahle DO Effect of high-intensity interval training in *de novo* heart transplant recipients in Scandinavia. Circulation. (2019) 139(19):2198–211. 10.1161/CIRCULATIONAHA.118.036747 30773030

[24] Rolid K Andreassen AK Yardley M Gude E Bjørkelund E Authen AR Long-term effects of high-intensity training vs moderate intensity training in heart transplant recipients: a 3-year follow-up study of the randomized-controlled HITTS study. Am J Transplant. (2020) 20(12):3538–49. 10.1111/ajt.16087 32484261

[25] Rolid K Andreassen AK Yardley M Gude E Bjørkelund E Authen AR High-intensity interval training and health-related quality of life in *de novo* heart transplant recipients—results from a randomized controlled trial. Health Qual Life Outcomes. (2020) 18(1):283. 10.1186/s12955-020-01536-4 32807179 PMC7433122

[26] Ulvestad M Durheim MT Kongerud JS Lund MB Edvardsen E. Effect of high-intensity training on peak oxygen uptake and muscular strength after lung transplantation: a randomized controlled trial. J Heart Lung Transplant. (2020) 39(9):859–67. 10.1016/j.healun.2020.06.006 32674956

[27] Ulvestad M Godang K Durheim MT Kongerud JS Brit Lund M Bollerslev J Effect of high-intensity training on bone health and body composition in lung transplant recipients: a secondary analysis of a randomized controlled trial. Clin Transplant. (2021) 35(8):e14375. 10.1111/ctr.14375 34048083

[28] Dall CH Snoer M Christensen S Monk-Hansen T Frederiksen M Gustafsson F Effect of high-intensity training versus moderate training on peak oxygen uptake and chronotropic response in heart transplant recipients: a randomized crossover trial. Am Transplant. (2014) 14(10):2391–9. 10.1111/ajt.12873 25135383

[29] Dall CH Gustafsson F Christensen SB Dela F Langberg H Prescott E. Effect of moderate- versus high-intensity exercise on vascular function, biomarkers and quality of life in heart transplant recipients: a randomized, crossover trial. J Heart Lung Transplant. (2015) 34(7):1053–2498. 10.1016/j.healun.2015.02.001 25840503

[30] Cappelle M Masschelein E Vos R Van Remoortel H Smets S Vanbekbergen J High-intensity training for 6 months safely, but only temporarily, improves exercise capacity in selected solid organ transplant recipients. Transplant Proc. (2021) 53(6):1836–45. 10.1016/j.transproceed.2021.03.040 34049699

[31] Rafique M Solberg OG Gullestad L Bendz B Holm NR Neghabat O A randomized clinical study using optical coherence tomography to evaluate the short-term effects of high-intensity interval training on cardiac allograft vasculopathy: a HITTS substudy. Clin Transplant. (2022) 36(1):e14488. 10.1111/ctr.14488 34747048

[32] Billany RE Smith AC Hutchinson GM Graham-Brown MPM Nixon DGD Bishop NC. Feasibility and acceptability of high-intensity interval training and moderate-intensity continuous training in kidney transplant recipients: the PACE-KD study. Pilot Feasibility Study. (2022) 8(1):106. 10.1186/s40814-022-01067-3 PMC9123685 35597974

[33] Hutchinson GM Cooper AM RE Nixon DGD Bishop NC Smith AC. Effect of high intensity interval training and moderate intensity continuous training on lymphoid, myeloid and inflammatory cells in kidney transplant recipients. Exerc Immunol Rev. (2022) 28:100–15. 35452395

[34] Masschelein E De Smet S Denhaerynck K Ceulemans LJ Monbaliu D De Geest S. Patient- reported outcomes evaluation and assessment of facilitators and barriers to physical activity in the transplantoux aerobic exercise intervention. PLoS One. (2022) 17(10):e0273497. 10.1371/journal.pone.0273497 36288368 PMC9605336

[35] Königsrainer I Löffler M Bühler S Walter M Schafbuch L Beckert S Impact of endotoxin exposure after exhausting exercise on the immune system in solid organ transplant recipients. Exerc Immunol Rev. (2012) 18. cited in: Ovid MEDLINE(R) at. Available online at: http://ovidsp.ovid.com/ovidweb.cgi?T=JS&PAGE=reference&D=med9&NEWS=N&AN=22876728 (accessed April 04, 2024). 22876728

[36] Cappuccilli M Mosconi G Roi GS De Fabritiis M Totti V Merni F Inflammatory and adipose response in solid organ transplant recipients after a marathon cycling race. Transplant Proc. (2016) 48(2):408–14. 10.1016/j.transproceed.2016.02.001 27109967

[37] Mosconi G Angelini ML Balzi W Totti V Roi GS Cappuccilli M Can solid-organ-transplanted patients perform a cycling marathon? Trends in kidney function parameters in comparison with healthy subjects. Transplant Proc. (2016) 48(2):415–9. 10.1016/j.transproceed.2015.12.042 27109968

[38] Schrutka L Slama A Muehlbacher J Bessa V Lichtenegger P Ghimessy Á Cardiopulmonary response to high-altitude mountaineering in lung transplant recipients-The Jebel Toubkal experience. Scand J Med Sci Sports. (2021) 31(10):1941–8. 10.1111/sms.14008 34170580

[39] Pirenne J Van Gelder F Kharkevitch T Nevens F Verslype C Peetermans WE Tolerance of liver transplant patients to strenuous physical activity in high-altitude. Am J Transplant. (2004) 4:554–60. 10.1111/j.1600-6143.2004.00363.x 15023147

[40] De Smet JM Niset G Degre S Primo G. Jogging after heart transplantation. N Engl J Med. (1983) 309(24):1521–2. 10.1056/NEJM198312153092415 (make AMA). 6358889

[41] Browne G Allan P Madhavan KK Winney R. Exercise-induced anuria in a renal allograft recipient. Nephrol Dial Transplant. (2001) 16(12):2431–3. 10.1093/ndt/16.12.2431 11733642

[42] Haykowsky M Tymchak W. Superior athletic performance two decades after cardiac transplantation. N Engl J Med. (2007) 356:2007–8. 10.1056/NEJMc070061 17494943

[43] Haykowsky MJ Riess KJ Baggish AL. Heart transplant recipient finishes the 118th Boston marathon 27 years post-surgery. J Heart Lung Transplant. (2014) 33(11):1197. 10.1016/j.healun.2014.07.013 25200379

[44] Haykowsky MJ Halle M Baggish A. Upper limits of aerobic power and performance in heart transplant recipients: legacy effect of prior endurance training. Circulation. (2018) 137(7):650–2. 10.1161/CIRCULATIONAHA.117.031445 29440194 PMC5815397

[45] Einollahi B Nafar M Taheri S Nemati E. Renal allograft in a professional boxer. Saudi J Kidney Dis Transpl. (2008) 19(2):241–3. 10.4239/sjcdt.2008.241 18310875

[46] Haykowsky M Taylor D Kim D Tymchak W. Exercise training improves aerobic capacity and skeletal muscle function in heart transplant recipients. Am J Transplant. (2009) 9(7):734–9. 10.1111/j.1600-6143.2008.02531.x 19344465

[47] Arora S. Immunological and non-immunological markers of cardiac allograft vasculopathy amongst heart transplant recipients (Thesis No. 934). University of Oslo, Oslo, Norway (2010).

[48] Ramzy D Rao V Brahm J Miriuka S Delgado D Ross HJ. Cardiac allograft vasculopathy: a review. Can J Surg. (2005) 48(4):319–27. 16149368 PMC3211528

[49] Henein MY Vancheri S Longo G Vancheri F. The role of inflammation in cardiovascular disease. Int J Mol Sci. (2022) 23(21):12906. 10.3390/ijms232112906 36361701 PMC9658900

[50] Nytrøen K Rustad LA Erikstad I Aukrust P Ueland T Lekva T Effect of high-intensity interval training on progression of cardiac allograft vasculopathy. J Heart Lung Transplant. (2013) 32(9):925–31. 10.1016/j.healun.2013.06.023 23906899

[51] Kubo T Akasaka T Shite J Suzuki T Uemura S Yu B OCT compared with IVUS in a coronary lesion assessment: the OPUS-CLASS study. JACC Cardiovasc Imag. (2013) 6(10):1095–104. 10.1016/j.jcmg.2013.04.014 24011777

[52] Franklin BA. Preventing exercise-related cardiovascular events: is a medical examination more urgent for physical activity or inactivity? Circulation. (2014) 129(10):1081–4. 10.1161/CIRCULATIONAHA.114.007641 24421369

[53] Eijsvogels TMH Thompson PD Franklin BA. The “extreme exercise hypothesis": recent findings and cardiovascular health implications. Curr Treat Options Cardiovasc Med. (2018) 20(10):84. 10.1007/s11936-018-0674-3 30155804 PMC6132728

[54] Barman PM VanWagner LB. Cardiac risk assessment in liver transplant candidates: current controversies and future directions. Hepatology. (2021) 73(6):2564–76. 10.1002/hep.31647 33219576 PMC8220582

[55] Nassar M Nso N Lakhdar S Kondaveeti R Buttar C Bhangoo H New onset hypertension after transplantation. World J Transplant. (2022) 12(3):42–54. 10.5500/wjt.v12.i3.42 35433331 PMC8968475

[56] Kourek C Karatzanos E Nanas S Karabinis A Dimopoulos S. Exercise training in heart transplantation. World J Transplant. (2021) 11(11):466–79. 10.5500/wjt.v11.i11.466 34868897 PMC8603635

[57] Thum JS Parsons G Whittle T Astorino TA. High-intensity interval training elicits higher enjoyment than moderate intensity continuous exercise. PLoS One. (2017) 12(1):e0166299. 10.1371/journal.pone.0166299 28076352 PMC5226715

[58] Leunis S Vandecruys M Cornelissen V Van Craenenbroeck AH De Geest S Monbaliu D Physical activity behaviour in solid organ transplant recipients: proposal ofTheory-driven physical activity interventions. Kidney and Dialysis. (2022) 2(2):298–329. 10.3390/kidneydial2020029

[59] Mathur S Levy RD Reid WD. Skeletal muscle strength and endurance in recipients of lung transplants. Cardiopulm Phys Ther J. (2008) 19(3):84–93. 10.1097/01823246-200819030-00003 20467503 PMC2845229

[60] Braith RW Conner JA Fulton MN Lisor CF Casey DP Howe KS Comparison of alendronate vs alendronate plus mechanical loading as prophylaxis for osteoporosis in lung transplant recipients: a pilot study. J Heart Lung Transplant. (2007) 26(2):132–7. 10.1016/j.healun.2006.11.004 17258146

[61] Nikkel LE Hollenbeak CS Fox EJ Uemura T Ghahramani N. Risk of fractures after renal transplantation in the United States. Transplantation. (2009) 87(12):1846–51. 10.1097/TP.0b013e3181a6bbda 19543063

[62] Lim WH Ng CH Ow ZGW Ho OTW Tay PWL Wong KL A systematic review and meta-analysis on the incidence of osteoporosis and fractures after liver transplant. Transpl Int. (2021) 34(6):1032–43. 10.1111/tri.13863 33835638

[63] WHO BMI Classification. Global Database on Body Mass Index. Available online at: http://www.who.int/bmi/index.jsp (accessed August 25, 2014).

[64] Milaniak I Dębska G Król B Wierzbicki K Przybyłowski P. Health locus of control and health behaviors in organ transplant recipients: a multicenter. Transplant Proc. (2022) 54(4):995–1001. 10.1016/j.transproceed.2022.02.058 35660277

[65] Yu H Zhao X Wu X Yang J Wang J Hou L. High-intensity interval training versus moderate-intensity continuous training on patient quality of life in cardiovascular disease: a systematic review and meta-analysis. Sci Rep. (2023) 13(1):13915. 10.1038/s41598-023-40589-5 37626066 PMC10457360

[66] Nieman DC Wentz LM. The compelling link between physical activity and the body’s defense system. J Sport Health Sci. (2019) 8(3):201–17. 10.1016/j.jshs.2018.09.009 31193280 PMC6523821

[67] Patel R Paya CV. Infections in solid-organ transplant recipients. Clin Microbiol Rev. (1997) 10(1):86–124. 10.1128/CMR.10.1.86 8993860 PMC172945

[68] Souza D Vale AF Silva A Araújo MAS de Paula Júnior CA de Lira CAB Acute and chronic effects of interval training on the immune system: a systematic review with meta-analysis. Biology (Basel). (2021) 10(9):868. 10.3390/biology10090868 34571745 PMC8465842

[69] Malthur S Janaudis-Ferreira T Wickerson L Singer LG Patcai J Rozenberg D Meeting report: consensus recommendations for a research agenda in exercise in solid organ transplantation. Am J Transplant. (2014) 14:2235e45. 25135579 10.1111/ajt.12874

[70] Wang B Li ZL Zhang YL Wen Y Gao YM Liu BC. Hypoxia and chronic kidney disease. EBioMedicine. (2022) 77:103942. 10.1016/j.ebiom.2022.103942 35290825 PMC8921539

[71] McKenzie KJ McKenzie DC Yoshida EM. Solid organ transplant recipients: clinical considerations in the application of exercise. BJSM Online. (2015) 49(2):76–8. 10.1136/bjsports-2014-093583 25115810

[72] Anselmi F Cavigli L Pagliaro A Valente S Valentini F Cameli M The importance of ventilatory thresholds to define aerobic exercise intensity in cardiac patients and healthy subjects. Scand J Med Sci Sports. (2021) 31(9):1796–808. 10.1111/sms.14007 34170582 PMC8456830

[73] Haykowsky MJ Riess KJ Schneider CA. Ironman triathlon performance pre- and post-heart transplant. J Heart Lung Transplant. (2015) 34(5):756. 10.1016/j.healun.2014.12.015 25727770

[74] Fink G Lebzelter J Blau C Klainman E Aravot D Kramer MR. The sky is the limit: exercise capacity 10 years post-heart-lung transplantation. Transplant Proc. (2000) 32(4):733–4. 10.1016/s0041-1345(00)00961-1 10856563

